# Cross-sectional study of correlation between mandibular incisor crowding and third molars in young Brazilians

**DOI:** 10.4317/medoral.18644

**Published:** 2013-02-05

**Authors:** Lilian H. Karasawa, Ana C. Rossi, Francisco C. Groppo, Felippe B. Prado, Paulo H F. Caria

**Affiliations:** 1 DDS, Department of Morphology, Anatomy area, Piracicaba Dental School, State University of Campinas - UNICAMP; 2 PhD student, Department of Morphology, Anatomy area, Piracicaba Dental School, State University of Campinas - UNICAMP; 3 Full Professor, Department of Physiological Sciences, Pharmacology/Anesthesiology/Therapeutics Area, State University of Campinas - UNICAMP, Piracicaba, SP, Brazil; 4 Assistant Professor, Department of Morphology, Anatomy area, Piracicaba Dental School, State University of Campinas - UNICAMP; 5 Associate Professor, Department of Morphology, Anatomy area, Piracicaba Dental School, State University of Campinas - UNICAMP

## Abstract

Objectives: The aim of this study was to evaluate transversally the clinical correlation between lower incisor crowding and mandible third molar. Study Design: Three hundred healthy volunteers (134 male and 166 female), aged 20.4 (±2.4) years-old were submitted to a complete clinical examination and filled up a questionnaire about gender, age, total teeth number and presence or absence of superior and inferior third molar. After a recent panoramic radiography were evaluated. The multiple logistic regression showed that none of the studied factors influenced the mandibular incisor crowding. Results: The proportion of both molars present or both absent was higher than the other conditions (Chi-square, p<.0001). The multiple logistic regression showed that any of the studied factors, influenced (p>.05) the mandibular incisor crowding. Despite the statistical significance, wear orthodontics appliances showed a little correlation (odds ratios < 1.0) in the mandibular incisor crowding. Conclusion: Presence of maxillary and/or mandibular third molars has no relation with the lower incisor crowding.

** Key words:**Malocclusion, third molars, lower incisor crowding, mandible.

## Introduction

Lower incisor crowding has been a subject of plenty discussions in the academic environment, especially concerning its etiology. The specialized literature has shown different opinions about this issue, attributing to the arch perimeter reduction as the main causal factor (1), other authors also point out the presence of third molar (2) or multiple factors (3).

A number of attempts have been performed in order to clarify this question. Many dentists still believe that the erupting third molars are able to push the anterior teeth forward causing anterior crowding (4). However, some studies relate no correlation between lower third molars and the lower incisor crowding (5).

The reduction of the lower dental arch perimeter caused by reduced interdental space, absence of interdental contacts and rota-tion/movement of teeth, generating dental crowding could be related to third molar (6). On the other hand longitudinal studies were unable to establish a relation between third molar and the mandibular dental crowding, considering subjects with unerupted, absent or extracted third molars (7). Despite of these studies, just one study mentioned the unilateral removal of mandibular third molar as a cause of small reduction in the degree of crowding at the extracted side (8).

Some authors attributed the incisor crowding to the pressure exerted by mandibular third molar (8), on the other hand others do not consider this pressure capable to cause this condition (1).

Despite of the absence of a clear relationship between the mandibular incisor crowding and third molars presence, extraction of those teeth has been performed in order to prevent abnormal orthodontic condition (4,8-11).

The aim of this study was to evaluate the relationship between the third molars presence and the mandibular incisor crowding in Brazilian young adults.

## Material and Methods

A cross-sectional study was achieved with 300 volunteers, being 134 men with mean age 20.4±2.40 years-old and 166 women with mean age 20.5±2.43 years-old. All of the volunteers presented good oral and general health. This study received the approval of the Local Research Ethic Committee.

All volunteers filled up a questionnaire regarding to age, gender, presence of upper and lower teeth, presence or absence of erupted third molar, presence or absence of both upper and lower premolars and mandibular incisor crowding. Data from the forms were confirmed by oral examination carried out by a calibrated operator and confirmed by a recent panoramic radiograph. The teeth not clinically visible and below the oral mucosa (confirmed by the radiograph) were considered unerupted.

Volunteers wearing prosthesis, showing absence of any teeth (except the third molars) or wearing fixed orthodontic appliances for any reason not related to mandibular incisor crowding, were excluded from the study.

Multiple logistic regression was used to analyze the relationship between mandibular incisor crowding and gender, upper and/or lower third-molar and/or premolars presence. In addition, Chi-squared test was used in order to observe the distribution of upper and lower third molars. The significance level was set at 5% for all tests and the statistical software was BioEstat 4.0 (Mamiraua Institute, Belem, PA, Brazil) for Windows.

## Results

The proportion of both molars present or both absent was higher than the other conditions (Chi-square, p<.0001) ( Table 1).

**Table 1:**
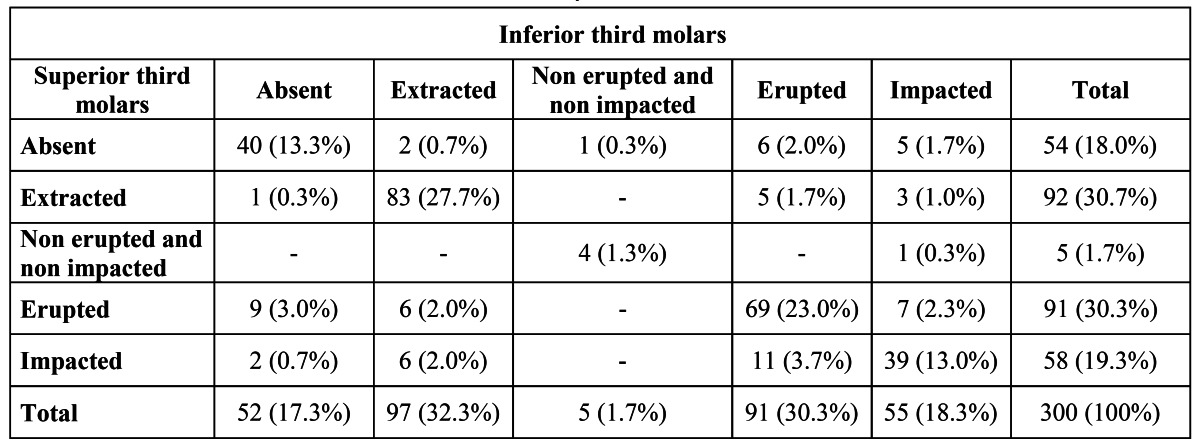
Distribution of the third molars observed on the study.

The distribution of the lower incisor crowding according to the lower and upper molars - lower incisor crowding and lower and upper third molars – correlation was founded ( Table 2).

**Table 2:**
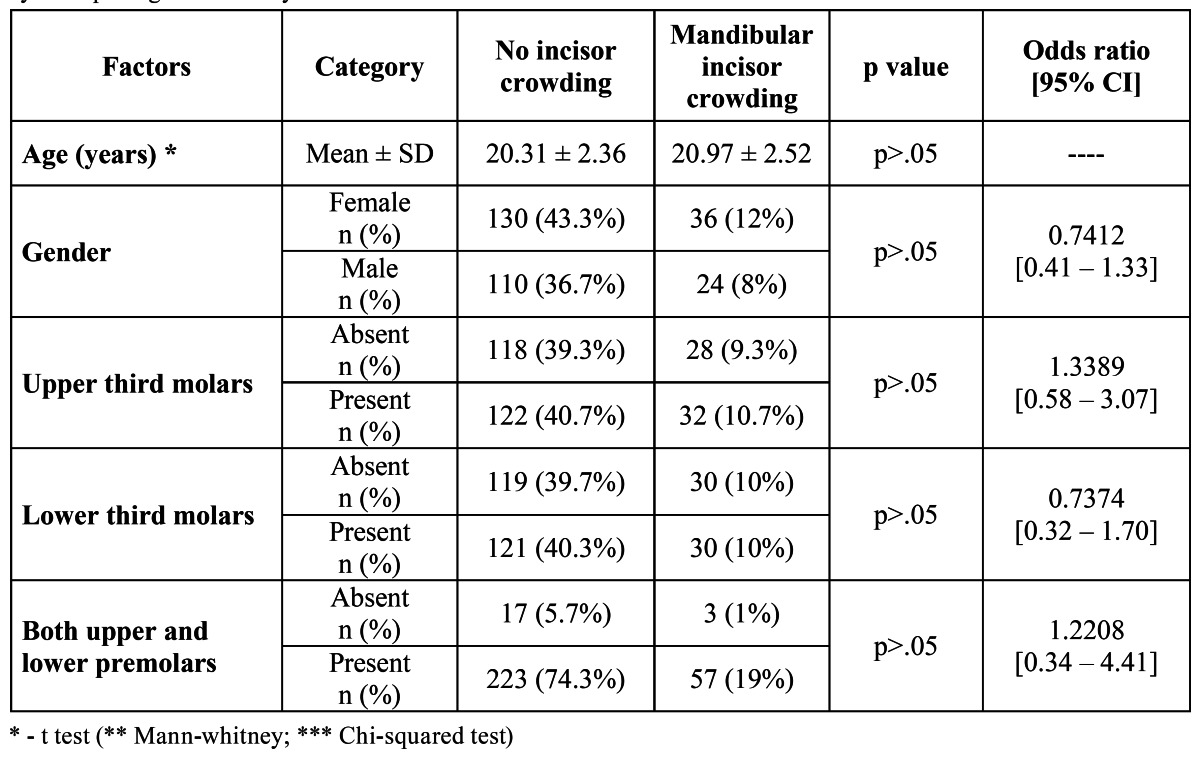
Distribution of the clinical characteristics and the relative risks for mandibular incisor crowding obtained by multiple regression analysis.

The multiple logistic regression showed that any of the studied factors, influenced (p>.05) the mandibular incisor crowding.

Despite the statistical significance, wear orthodontics appliances also showed a little correlation (odds ratios < 1.0) in the mandibular incisor crowding. Upper and/or lower third molar and/or premolars presence and orthodontics appliances wearing.

## Discussion

The results of the present study did not present correlation between third molars and lower incisor crowding. This question is controversial because although previous studies related relationship between those conditions (2,6,8,10-13) and many professionals still believe that eruption of third molar exerts force on the dental arch causing dental crowding, therefore recommend the prophylactic extraction of third molars (14,15).

It is important to consider that many associated factors may cause lower incisor crowding such as decrease the length of the dental arc after the second molar eruption (4,9,13,14,16), as well as inter-canine distance (2,4,16). The action of multiple factors at different stages of cranial development may contribute to the lower crowding. The pressure on the back of the dental arch due to physiological mesial slide; the anterior component of occlusal force on mesially inclined teeth, the mesial vector due to muscle contraction, or the presence of the third molar development can cause forward movement of the posterior teeth and therefore shortening the dental arc and increase the crowding (12). The influence of late mandibular development (8) provoking changes in the complex growth pattern can also result in anterior crowding (7), changes in the muscular function of the cheeks, lips and tongue can change the dental balance (9) and mouth breathing also may cause muscular unbalance and provoke lower incisor crowding.

Follow up studies and measurements of dental models seem to better evaluate the possible correlation between presence of third molars and lower incisor crowding (1,7,8,17,18). However, neither method used to analyze those conditions was able to confirm any correlation between them.

Research design in longitudinal studies like contact maintenance, variable localization, loss of interest by the volunteers during the research (1) are some of the critical aspects of the longitudinal studies. Cross-sectional studies like the present study can also be considered a satisfactory evaluation to observe the variables studied because do not present limitations as the long term studies, can analyze the occurrence of events at the same time and its possible correlations.

We recognize that a simple observation of dental crowding and third molars presence is not enough to figure out the complexity of possible interaction of factors like: facial growth pattern; tooth size and arch form; cumulative effects of resting; continuing late growth rotations, functional and parafunctional soft tissue pressures; lack of compensating attrition and mesially acting force from the back of the dental arch (19). Nevertheless, remains unknown the degree of interaction of those factors and how to analyze them in vivo.

Different statistical tests have been used to evaluate the relationship between lower incisor crowding and third molars presence (3,13,16). When statistically significant differences are mentioned, the clinical significance of this information remains controversial, because is difficult to establish the precise lower incisor crowding level to define the third molar extraction as a preventive procedure (7,8).

On the present study when the maxillary third molars were extracted the mandibular ones were also removed (or vice versa). We considered this fact, along with the absence of any relation between the dental crowding and maxillary and/or mandibular third molars presence, a clearly indication that the extraction of third molars is not a good way to prevent the mandibular incisors crowding (16).

However, arguments like the strength created by mandibular third molars could cause inferior dental crowding along with buc-cal-lingual movement of the inferior second molars have been used to justify the third molars extraction (13).

Similar results of this study were also presented by Vasir and Robinson (2) that did not observe any correlation between inferior dental crowding and third molars presence. These authors considered as etiologic factors the inferior dental crowding growth and remodeling of mandible; preexisting discrepancy among dental tissues; occlusal disturbances; the size and shape of teeth; soft tissue development; and also the third molars presence.

The third molar removal is one of the most common surgical procedures performed. The prophylactic removal of asymptomatic impacted third molar is defined as the (surgical) removal of third molar in the absence of local disease. Little controversy surrounds the removal of impacted third molars when they are associated with pathological changes such as infection, non-restorable carious lesions, cysts, tumors, inflammation of the gums around the tooth and destruction of adjacent teeth and bone. Several other reasons to justify prophylactic removal have also been given (18,19).

However, the justification for prophylactic removal of impacted third molars is less certain and has been debated for many years. Song et al. (18) reviewed the literature about this subject and mentioned nine reviews that considered a weak association between retention of third molars and anterior crowding. Six out of 21 reviews with a more general scope also concluded that the prophylactic removal of third molars was unjustified. Twelve general reviews did not conclude with a clear message about the management of third molars. Three reviews suggested that prophylactic removal of third molars is appropriate, but these reviews have used poorer methodological criteria than the previous. Three out of four papers were focused on surgical management.

Surgical removal carried out in older patients increases the risk of more postoperative complications. However, in most developed countries the prophylactic removal of third molar, impacted or fully erupted, has been considered appropriate. The observance of specified indicators for dental removal may reduce the number of surgical procedures by 60% or more. It has been suggested that careful monitoring of asymptomatic third molars may be an appropriate strategy. No evidence was found to support or refute the prophylactic removal of asymptomatic impacted third molars in adults. There is some reliable evidence that suggests that the prophylactic removal of asymptomatic impacted third molar in adolescents neither reduces nor prevents late incisor crowding (19).

Although many theories have attempted to explain the reasons of the lower incisor crowding, many factors correlated or not among them, can be responsible for that dental condition. The present study does not provide enough clinical evidences about third molars as the etiologic factor in the late lower dental arch crowding.
